# Platelets camouflaged nanovehicle improved bladder cancer immunotherapy by triggering pyroptosis

**DOI:** 10.7150/thno.99040

**Published:** 2024-10-14

**Authors:** Jiale Tian, Mingde Gao, Jinfeng Zhu, Haifei Xu, Hao Ji, Donglin Xia, Xiaolin Wang

**Affiliations:** Nantong Tumor Hospital, Affiliated Tumor Hospital of Nantong University & School of Public Health of Nantong University, Nantong, 226000, P. R. China

**Keywords:** pyroptosis, immunotherapy, target delivery, nanovehicle, bladder cancer

## Abstract

The regulation of immunosuppressive microenvironments in tumors through targeted drug delivery shows promise for immunochemotherapy in bladder cancer. Drawing inspiration from stealth tactics, a nano-vehicle camouflaged with platelets (PLTs) was developed to enable precise delivery and trigger pyroptosis for tumor immunotherapy.

**Methods:** Erdafitinib (Erda) was nano-sized and encapsulated in PLTs to construct nano-Erda@PLT. Characterization of the PLTs camouflaged nano-vehicle was conducted using Zetasizer, SEM, and confocal laser scanning microscopy. The excellent targeted delivery property of the PLTs nano-vehicle was investigated through intravital imaging, three-dimensional microspheres, and SEM. Validation of pyroptosis in bladder cancer cells via the caspase-3/GSDME pathway was performed using western blot, immunofluorescence, and ELISA tests. Immunotherapy by nano-Erda@PLT treatment *in vivo* was confirmed using H&E, immunohistochemical, and flow cytometry. Lastly, the side effects of nano-Erda@PLT were assessed.

**Results:** Proteomic analysis revealed that the activation of p-selectin on platelets facilitated the identification of nano-Erda@PLT targeted therapies. Nanoscale of Erda released in response to adenosine diphosphate, facilitated intratumoral permeation. This could contribute to an upregulation of the key proteins of pyroptosis, caspase-3 and GSDME, in bladder cancer cells due to nano-Erda@PLT accumulation. Additionally, the burst release of numerous inflammatory factors may enhance the system's adaptive immune response. In a bladder cancer animal model, this treatment was found to regulate the immunosuppressive microenvironment, resulting in effective tumor immunotherapy and the induction of a long-lasting, robust immune memory.

**Conclusion:** PLTs-camouflaged nano-vehicles enable nano-Erda-mediated tumor immunotherapy through the induction of pyroptosis. These findings introduce a novel approach in exploring nanomaterial-mediated pyroptosis for cancer immunotherapy.

## Introduction

The advent of immunotherapy has brought new hope to cancer patients by enhancing the immune system's ability to target cancer cells 1,2. Immunotherapies that focus on CD4^+^ and CD8^+^ T cells have shown great potential in treating various cancers, including bladder cancer 3-5. In the realm of immunotherapy, CD4^+^ and CD8^+^ T cells are crucial for identifying and eliminating tumors. CD4^+^ T cells assist in activating CD8^+^ T cells, which then transform into effector cells capable of directly destroying cancer cells 6. Researchers are currently exploring ways to boost the activity of CD4^+^ and CD8^+^ T cells to enhance the effectiveness of immunotherapy. Overall, these T cells play a vital role in the immune response against tumors and are integral to the success of immunotherapy 7. By delving into the biology of these cells and devising strategies to enhance their function, we can enhance the efficacy of immunotherapy and improve outcomes for cancer patients.

Pyroptosis is recognized as an immunogenic form of cell death, eliciting an immune response 8,9. Numerous studies have demonstrated that pyroptosis can boost the effectiveness of T cell-based immunotherapies, such as adoptive cell therapy and immune checkpoint blockade 10-14. This enhancement is attributed to the release of pro-inflammatory cytokines and tumor antigens, which can activate antigen-presenting cells (APCs) and T cells 15-17. By augmenting T cell function, pyroptosis has the potential to enhance the efficacy of T cell-based immunotherapies and yield improved outcomes for cancer patients. However, inducing pyroptosis in cancer cells poses a significant challenge, as these cells often develop resistance to cell death pathways 18-20. Despite these obstacles, researchers are actively exploring strategies to induce pyroptosis in cancer cells, including the development of drugs targeting pyroptosis-related proteins, which could potentially overcome resistance to pyroptosis induction 21-26. Therefore, inducing pyroptosis in cancer cells presents a challenging yet promising approach for enhancing the efficacy of cancer immunotherapy.

Enhancing the therapeutic effectiveness of chemotherapeutic drugs through targeted delivery and increased accumulation at the tumor site is a crucial goal 27. High-dose chemotherapy can trigger cytokine release syndrome via pyroptosis 28,29, underscoring the importance of precise delivery of chemotherapeutics to the tumor in both preclinical and clinical settings. Erdafitinib (Erda) has demonstrated promising results in treating urothelial carcinoma patients with FGFR alterations 30-32, particularly FGFR2 and FGFR3 mutations, which are common in this type of cancer and linked to poor prognosis. However, systemic administration of the medication may not rapidly achieve the necessary local concentration for inducing pyroptosis in tumor cells due to unrestricted distribution in the body. Therefore, identifying an effective targeted carrier for swift drug delivery to bladder cancer represents a promising strategy for inducing pyroptosis in bladder cancer.

**Scheme 1:**
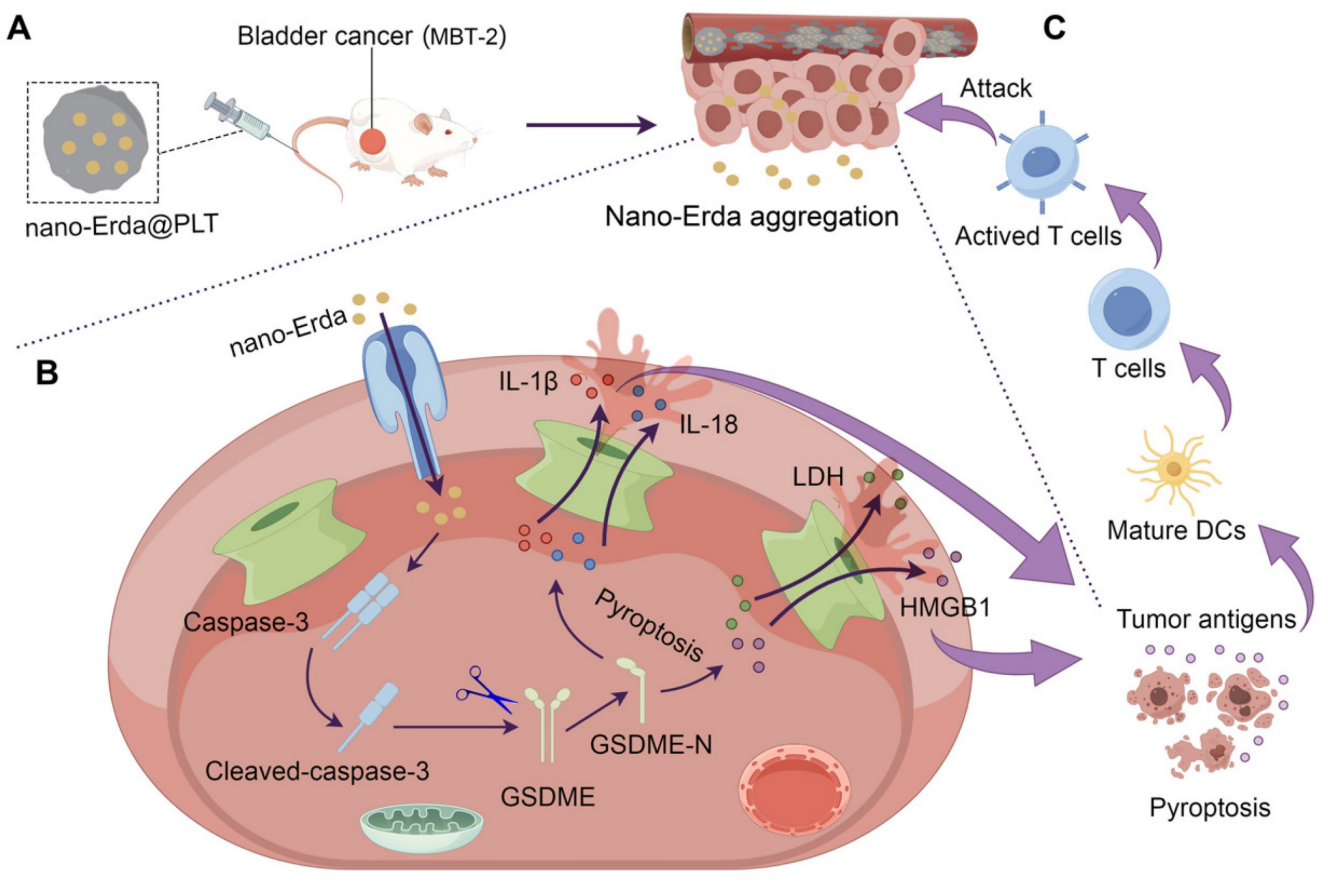
Schematic illustration of PLTs camouflaged nanovehicle for nano-Erda (nano-Erda@PLT) for improving bladder cancer immunotherapy by triggering pyroptosis. (A) Nano-Erda@PLT was administered intravenously into bladder cancer bearing model mice. PLTs camouflaged nanovehicle released nano-Erda particles upon activation at the bladder tumor, ensuring drug aggregation. (B) Aggregated nano-Erda induced pyroptosis through the caspase-3/GSDME pathway. Nano-Erda triggered the cleavage of caspase-3, leading to specific cleavage of GSDME. The cleaved GSDME-N fragment forms pores on the cell membrane, resulting in the release of inflammatory factors, such as lactate dehydrogenase (LDH), high mobility group box 1 (HMGB1) protein, interleukin-1 beta (IL-1β), and interleukin-18 (IL-18). (C) Inflammatory factors lead to rapid and extensive pyroptosis of neighboring bladder cancer cells, releasing a large amount of tumor antigens. These antigens were then taken up by dendritic cells and presented to T cells, ultimately leading to T cell attack and clearance of tumor cells.

Platelets (PLTs) are specialized blood cells that have the unique ability to actively home in on sites of vascular injury and inflammation, making them an ideal choice for delivering drugs to specific tissues or organs 36,37. When loaded with drugs, PLTs can travel through the circulatory system until they reach their intended destinations 38,39. PLTs possess the capability to release their payload upon activation, which is triggered by factors present at the target site, ensuring precise and controlled drug release 40. Inspired by this, this study utilized a PLTs-camouflaged nanovehicle as a novel drug delivery system for nano-Erda (nano-Erda@PLT) to enhance targeted delivery to bladder tumors, inducing tumor pyroptosis and subsequently boosting immune response (Scheme 1A). Through targeted delivery, nano-Erda accumulated at the bladder cancer site, due to the PLTs-camouflaged nanovehicle of tumor-specific delivery. This accumulation then triggered caspase-3, resulting in GSDME cleavage and subsequent pyroptosis of bladder cancer cells (Scheme 1B). Following pyroptosis, the release of a substantial amount of inflammatory factors stimulated T cells, thereby enhancing immune response (Scheme 1C). By leveraging the inherent targeting abilities of PLTs, this approach opens up new avenues for immune therapy and offers a promising strategy for drug delivery in the treatment of bladder cancer.

## Methods

### Materials and reagents

Erdafitinib (Erda) (BBM0888, Biobomei), Cysteamine (BC9344, Biobomei), Prostaglandin E1 (PGE1) (QP5828, Biobomei) and Adenosine-5'-Diphosphate (ADP) (XA6584, Biobomei) was used in the construction and activation of nano-Erda@PLT.

Primary antibodies used in the Western blot experiments: anti-GSDME-N (Abcam, AB215191), anti-caspase-3 (Bioss, bs-0081R), anti-CD41 (Proteintech, 24552-1-AP), anti-CD61 (Proteintech, 18309-1-AP), anti-CD62p (Bioss, bs-0561R), anti-GAPDH (Proteintech, 60004-1-Ig) and anti-β-actin (Proteintech, HRP-66009). Secondary HRP-conjugated antibodies used were anti-mouse IgG and anti-rabbit IgG (Proteintech). Primary antibodies used in the immunofluorescence and immunohistochemistry experiments: anti-caspase-3 (Bioss, bs-0081R), anti-GSDME (Affinity, DF9705) and anti-CD62p (Bioss, bs-0561R). Secondary antibody used in the immunofluorescence experiments was SAlexa Fluor 594-anti-rabbit IgG (Solarbio, K1034G-AF594). Secondary antibody used in the immunohistochemistry experiments was HRP-anti-rabbit IgG (Servicebio, GB23303). Antibodies used in the flow cytometry experiments: FITC anti-CD3 (BioLegend, 100204), PE anti-CD8 (BioLegend, 100708), PE/Cyanine7 anti-IFN-γ (BioLegend, 505826), PE anti-CD4 (BioLegend, 100512), PE/Cyanine7 anti-CD25 (BioLegend, 102016), FITC anti-CD11c (BioLegend, 117306), PE anti-CD40 (BioLegend, 157506), PE anti-CD86 (BioLegend, 159204), PE anti-MHC-II (Proteintech, PE-65122) and FITC anti-CD19 (BioLegend, 115506). Annexin V-Alexa Fluor 488/PI Apoptosis Assay Kit (FcMACS, FMSAV488) was used in flow cytometry to detect cellular pyroptosis.

### Cells and animals

MBT-2 cells (murine bladder cancer cells) were sourced from Haixing Biosciences in Suzhou, China, while bEnd.3 mouse normal vascular endothelial cells were acquired from iCell Bioscience Inc. in Shanghai, China. The cells were cultured in RPMI-1640 or DMEM with 10% FBS and 1% penicillin/streptomycin at 37 °C in a 95% air and 5% CO_2_ environment. The culture medium was refreshed or cells were passaged every 2-3 days at a split ratio of 1:2-1:4.

ICR and C3H mice, both aged 4 weeks and of equal numbers of male and female, were obtained from the Animal Center of Nantong University and SPF Biotechnology Co., Ltd. in Beijing, China, respectively. Prior to subcutaneous tumor experiments, the mice were acclimated for 1 week. Subsequently, MBT-2 cells were subcutaneously injected into the leg root of each mouse at a concentration of 5×10^6^ cells/100 μL suspension per mouse. All animal experiments were conducted following guidelines for the care and use of laboratory animals and were approved by the Institutional Animal Ethics Committee.

### Construction and characterization of nano-Erda@PLT

Erda underwent surface modification by activating carboxyl groups on drug molecules, followed by the addition of cysteamine post ultrafiltration. The cysteamine-modified Erda was then dissolved in dimethyl sulfoxide (DMSO), thoroughly stirred, and membrane-filtered to obtain a nano-Erda solution. Fresh blood was collected from Sprague-Dawley rats (approximately 200 g) and processed to obtain a PLT-rich plasma solution via gradient centrifugation. To prevent the activation of PLT during the extraction process, 1 μM PGE1 was added. The nano-Erda solution was mixed with the PLT solution and subjected to ultrasound at room temperature with a power of 100 W for a minimum of 6 minutes using a bath sonicator to create the nano-Erda@PLT solution.

Particle size and surface potential changes during the construction of nano-Erda@PLT were determined using a Zetasizer Nano ZS90 (Malvern, UK). Scanning electron microscopy (SEM) images were captured using a GeminiSEM 300 microscope (ZEISS, Germany) to analyze the morphological changes of nano-Erda@PLT during the preparation process. PLT and nano-Erda@PLT samples were fixed overnight in phosphate buffer solution (PBS) containing 2.5% (v/v) glutaraldehyde. Dehydration was carried out using a gradient of ethanol concentrations - 35%, 70%, 80%, 95%, and 100%. The samples were then deposited onto silicon slides, dried, coated with a 10 nm thick gold film, and observed via SEM.

Confocal laser scanning microscopy was utilized to characterize and demonstrate the successful encapsulation of nano-Erda within PLTs. Rhodamine B underwent chemical modification by introducing reactive amino/ carboxyl groups. It was then mixed with nano-Erda solution at a molar ratio of 5:1 (Rhodamine B to nano-Erda) and the labeling process was carried out in a dark environment for a minimum of 30 minutes. Afterward, the mixture was dialyzed in PBS for 12 hours to remove the free rhodamine B. Combined FITC with PLTs in a ratio of 500 μM to 10^5^, then placed the mixture in a refrigerator at 4 °C and stired continuously for 12 to 18 hours. Dialyzed the solution to remove any unbound FITC, resulting in FITC-labeled PLTs. Ultrasound was applied at a power setting of 100 W for over 6 minutes to load nano-Erda into PLTs, resulting in fluorescently labeled nano-Erda@PLT. The fluorescently labeled nano-Erda@PLT were visualized using a Leica TCS SP8 STED 3X microscope (Wetzlar, Germany).

### Immunofluorescence

Cells were fixed at room temperature for 15 minutes with 4% paraformaldehyde, followed by the addition of Triton X-100 for 20 minutes to permeabilize the cell membrane. Subsequently, 5% BSA (bovine serum albumin) was applied as blocking agent at 37 ℃ for 1 hour to reduce nonspecific binding of antibodies. Primary antibodies were introduced and left to incubate overnight at 4 ℃. The next day, secondary antibodies were added and incubated at 37 °C for 1 hour. DAPI staining of cell nuclei was achieved by a 3-minute exposure. Finally, the samples were promptly examined under a fluorescence microscope.

### Immunohistochemistry

Following deparaffinization, dehydration, antigen retrieval, endogenous peroxidase blocking, and blocking with 3% BSA serum, the paraffin sections were exposed to the primary antibody overnight at 4 °C. Subsequently, they underwent incubation with HRP-labeled secondary antibody at room temperature for 50 minutes, followed by DAB staining. Counterstaining with hematoxylin was carried out to visualize the cell nuclei. After dehydration and mounting, the sections were examined under a microscope.

### Western blot analysis

Proteins from the cells and platelets were prepared using standard protocols. Following determination of the protein concentration, the protein solution was denatured in a constant temperature water bath at 97 °C for 5-10 minutes. Subsequently, protein samples were subjected to gel electrophoresis (Beyotime) and then transferred to a methanol-activated PVDF membrane (Millipore). The membrane was blocked with 5% skim milk-TBST for 120 minutes, followed by overnight incubation with primary antibodies at 4 °C. The next day, the membrane was incubated with secondary antibodies at room temperature for 60 minutes. Finally, the signals were detected using the Tanon-5200 fully automated chemiluminescence imaging system (Tanon, China).

### Flow cytometry

Single-cell suspension was prepared following the standard protocol, with the cell concentration diluted to approximately 1×10^6^ cells per 100 μL using a cell counter. Subsequently, suitable antibodies were added as per the flow cytometry antibody instructions, gently mixed, and incubated at 4 °C in the dark. Following two washes with pre-chilled PBS, the cells were promptly analyzed using the Attune NxT Acoustic Focusing Cytometer 4486515 (Thermofisher, USA) to determine the proportion of cells labeled with various fluorescent markers in each channel.

### Enzyme-linked immunosorbent assay (ELISA)

ELISA assay kits were employed to measure the levels of cytokines in the cell culture supernatant, specifically utilizing Mouse IL-1β ELISA Kit (Beyotime, PI301), Mouse IL-18 ELISA Kit (Beyotime, PI553), Mouse LDH ELISA Kit (Biobomei, ELS-1715), and Mouse HMGB1 ELISA Kit (Biobomei, ELS-2168), following the provided manufacturer's instructions.

### *In vitro* experiment on tumor targeting and transvascular effects of nano-Erda@PLT

MBT-2 mouse bladder cancer cells and bEnd.3 mouse normal vascular endothelial cells were cultured in 24-well plates until reaching 70-80% cell density. Following removal of the culture medium, cells were washed with PBS and fresh medium added. Subsequently, a Transwell chamber with a 0.4 μm pore size was placed on the cells, and fluorescently labeled nano-Erda@PLT (Erda labeled with rhodamine) was introduced into the chamber. After a 24-hour incubation period, the Transwell chamber was removed, and cells were washed with PBS before staining with DAPI. Following incubation and washing, the cells were immediately examined under a fluorescent microscope.

### *In vitro* experiment on tumor permeability of nano-Erda@PLT

MBT-2 mouse bladder cancer cells were cultured as spheroids on a 3-D cell culture plate with approximately 100 cells seeded per well. Medium replacement was performed every 3 days under careful observation using microscopy. After around 10 days, tumor spheroids were detected. Nano-Erda@PLT and free nano-Erda (rhodamine-labeled Erda, FITC-labeled PLT) were then introduced separately. Following a 24-hour incubation period, the supernatant was meticulously removed under microscopy prior to examination using a confocal laser scanning microscopy.

### *In vivo* experiment on tumor targeting of nano-Erda@PLT

Nano-Erda@PLT and nano-Erda, both fluorescently labeled with Cy5.5, were injected separately into the tail veins of ICR mice (5 weeks old, mixed genders) one week after subcutaneous injection of MBT-2 cells at the root of their thighs. The dosage is 0.1 mL injected into each animal, with a drug concentration of nano-Erda (3.0 mM), or nano-Erda@PLT (containing 300 × 10⁶/mL PLTs and 3.0 mM nano-Erda).

After 0.5 hours post-injection, the mice were anesthetized with isoflurane/O_2_ (2% v/v) and placed in the *in vivo* imaging system to observe the distribution of drug fluorescence. Subsequently, euthanasia was performed via CO_2_ asphyxiation, followed by dissection of the hearts, livers, kidneys, and tumor tissues of the mice to observe the fluorescence distribution of the drugs. The fluorescence was captured using an imaging system (IVIS Lumina Series III, PerkinElmer, USA) and analyzed with living image V4.4.

### *In vivo* immunotherapy efficacy and histological analysis

Tumor-bearing mice were randomly assigned to four groups when tumors reached approximately 50 mm³. The groups included a PBS control group and three treatment groups: PLT (300 × 10⁶/mL), nano-Erda (3.0 mM), and nano-Erda@PLT (containing 300 × 10⁶/mL PLTs and 3.0 mM nano-Erda). All solutions were administered via tail vein injection (volume: 100 μL). Tumor size was measured every other day, and tumor weight was assessed at the end of the study. For histological analysis, tumors were harvested and stained with hematoxylin and eosin (H&E), caspase-3, and GSDME. Additionally, tumor tissues from each group were collected for flow cytometry analysis to quantify immune cell populations.

### Biosafety assessment

C3H mice of mixed genders were implanted with MBT-2 cells subcutaneously at 5 weeks of age (day 0). Starting from the 7th day, they were administered intravenous injections of nano-Erda@PLT every 3 days, with a dosage of 300 nmol of nano-Erda per injection, totaling 5 treatments. Peripheral blood samples were collected weekly (on day 0, 8, 15, and 22) to obtain serum, which was then analyzed using a biochemical analyzer to measure alanine aminotransferase (ALT), aspartate aminotransferase (AST), creatine kinase (CK), blood urea nitrogen (BUN), creatinine (CREA), lactate dehydrogenase (LDH), and other parameters. Simultaneously, the mice were euthanized for autopsy, and vital organs such as the heart, liver, kidneys, and spleen were dissected and subjected to H&E staining for observation of potential toxic side effects.

### Statistical analysis

All statistical data were derived from a minimum of three independent experiments and presented as mean ± standard deviation (SD). Statistical analysis and plotting were performed using GraphPad Prism 10.2.2. Student's t-test was used to determine statistical differences between two groups, while analysis of variance (ANOVA) was used for differences among three or more groups. In all cases, a p-value of less than 0.05 was considered statistically significant, denoted as *P < 0.05, **P < 0.01, ***P < 0.001, ****P < 0.0001.

## Results, Discussion and Conclusions

### The concentration-dependent induction of pyroptosis by nano-Erda

**Figure 1:**
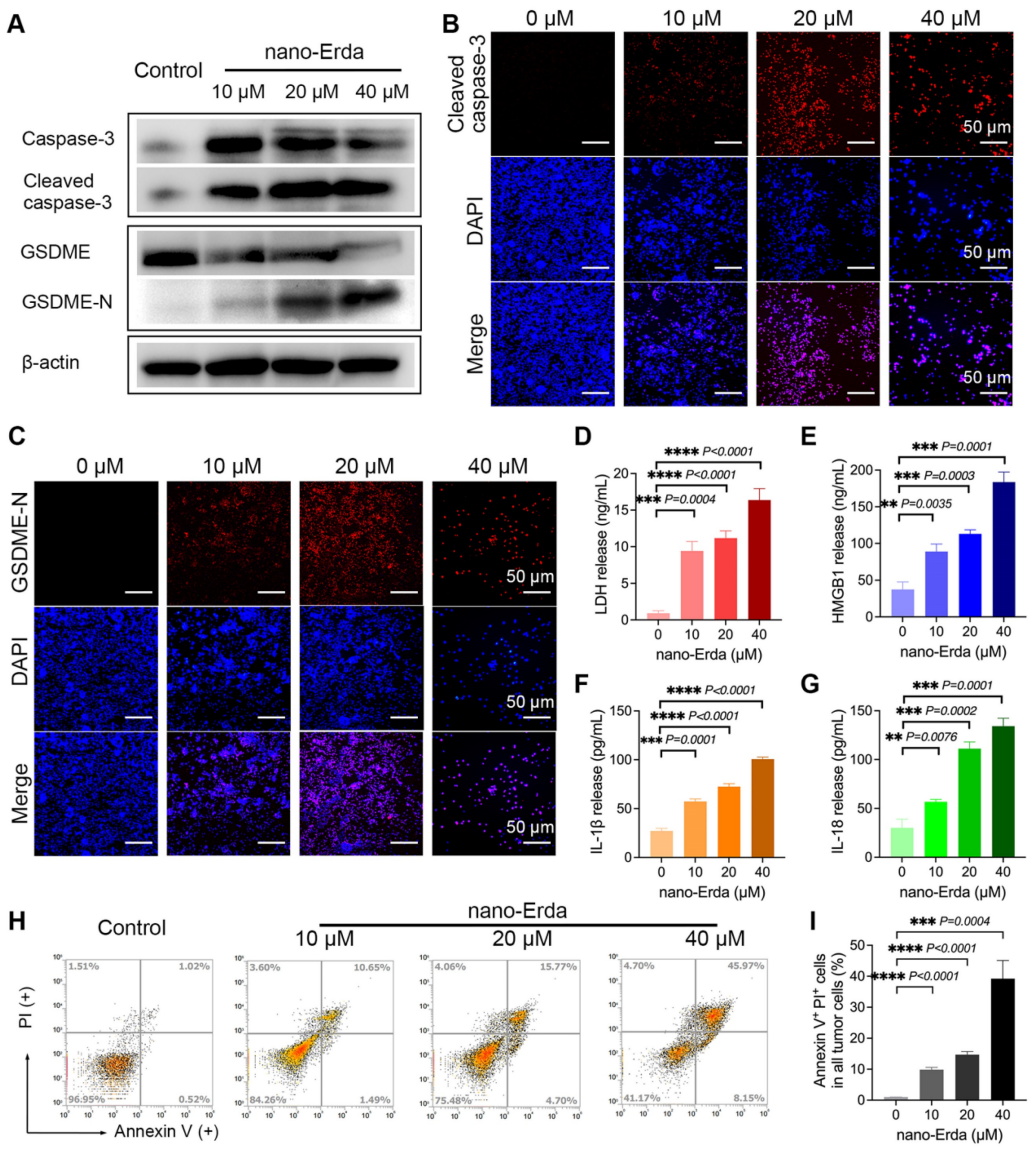
The concentration-dependent induction of pyroptosis by nano-Erda. (A) Protein expression of key pyroptotic molecules, including caspase-3, cleaved caspase-3, GSDME (full-length GSDME), and GSDME-N (N-terminal-cleaved GSDME). The MBT-2 cells were treated with concentrations of 0, 10, 20, and 40 μM of nano-Erda for 24 hours. (B) Immunofluorescence detection of cleaved caspase-3 after the MBT-2 cells were treated with concentrations of nano-Erda. (C) Immunofluorescence detection of GSDME-N. (D) The concentrations of LDH released from MBT-2 cells treated with different concentrations of nano-Erda for 24 h. (E) The concentrations of HMGB1 released from MBT-2 cells. (F) The concentrations of IL-1β released from MBT-2 cells. (G) The concentrations of IL-18 released from MBT-2 cells. (H) Representative flow cytometry results of nano-Erda treated MBT-2 cells, which are Annexin V^-^PI^-^ (LL), Annexin V^+^PI^-^ (LR), Annexin V^-^PI^+^ (UL), or Annexin V^+^PI^+^ (UR). (I) viability (% of Annexin V^+^PI^+^ cells). *Significantly different compared to control (*P* < 0.05). **P < 0.01, ***P < 0.001, ****P < 0.0001.

Pyroptosis is a crucial mechanism for eliminating damaged or unwanted cells, including cancer cells. However, not all drugs have the capability to induce pyroptosis in cancer cells 41. The ability of a drug to induce pyroptosis in cancer cells is influenced by various factors, such as its mechanism of action, the type and context of the cancer cells, drug dosage and exposure time, and acquired resistance 42. In this study, we investigated whether nano-Erda could induce pyroptosis in bladder cancer cells (MBT-2). Previous studies have shown that cell pyroptosis can occur through different pathways, including the caspase-3 and gasdermin E (GSDME) mediated pathways, which are essential for understanding programmed cell death 8,43. Upon the addition of nano-Erda to well-growing MBT-2 cells for 24 hours, key proteins in the pyroptosis pathway, such as cleaved caspase-3 and GSDME-N (N-terminal-cleaved GSDME), showed a significant increase (Figures 1A). This was due to nano-Erda triggered the activation and cleavage of caspase-3. Activated caspase-3 subsequently cleaved GSDME, resulting in the release of the GSDME-N fragment. GSDME-N perforates the cell membrane, leading to cell lysis and pyroptosis. As drug concentration increased, caspase-3 expression initially increased but then gradually decreased due to its cleavage into cleaved caspase-3. Cleaved caspase-3 expression continued to increase with increasing drug concentration. GSDME expression decreased as it was cleaved, while GSDME-N expression increased.

Interestingly, a concentration-dependent induction of pyroptosis by nano-Erda was observed, with the protein expression of cleaved caspase-3 and GSDME-N increasing significantly with higher concentrations of nano-Erda (Figure S1 and S2). Immunofluorescence analysis yielded similar results, with the fluorescence intensity of cleaved caspase-3 and GSDME-N increasing at higher concentrations of nano-Erda (Figure 1B-C, S3 and S4).

Pyroptosis is a tightly regulated process that encompasses a series of distinct events, including the release of specific cellular components like lactate dehydrogenase (LDH), high mobility group box 1 (HMGB1) protein, interleukin-1 beta (IL-1β), and interleukin-18 (IL-18) 44,45. The secretion of these factors was detected and illustrated in Figures 1D-G. The levels of pyroptosis-related cell factors significantly increased with nano-Erda concentration. Subsequently, flow cytometry revealed a rise in the percentage of cells undergoing pyroptosis. As depicted in Figure 1H, the percentage of Annexin-V^+^ PI^+^ cells, representing pyroptotic bladder cancer cells, gradually increased with higher concentrations of nano-Erda. Notably, at a concentration of 40 μM, nano-Erda induced a large amount of cell death. Quantitative analysis in Figure 1I further supported the induction of pyroptosis in bladder cancer cells by nano-Erda in a concentration-dependent manner.

Encouraged by our above *in vitr*o findings, the pyroptotic effects of nano-Erda in mice bearing bladder tumors were investigated. After five treatments, immunohistochemical analysis of caspase-3 and GSDME showed weak caspase-3 activation but no pyroptosis in tumor cells (Figure S5). This discrepancy between *in vitro* and *in vivo* outcomes highlighted the challenge of achieving tumor enrichment with nano-Erda via intravenous administration, emphasizing the need for targeted delivery strategies.

### Construction and characterization of nano-Erda@PLT

**Figure 2:**
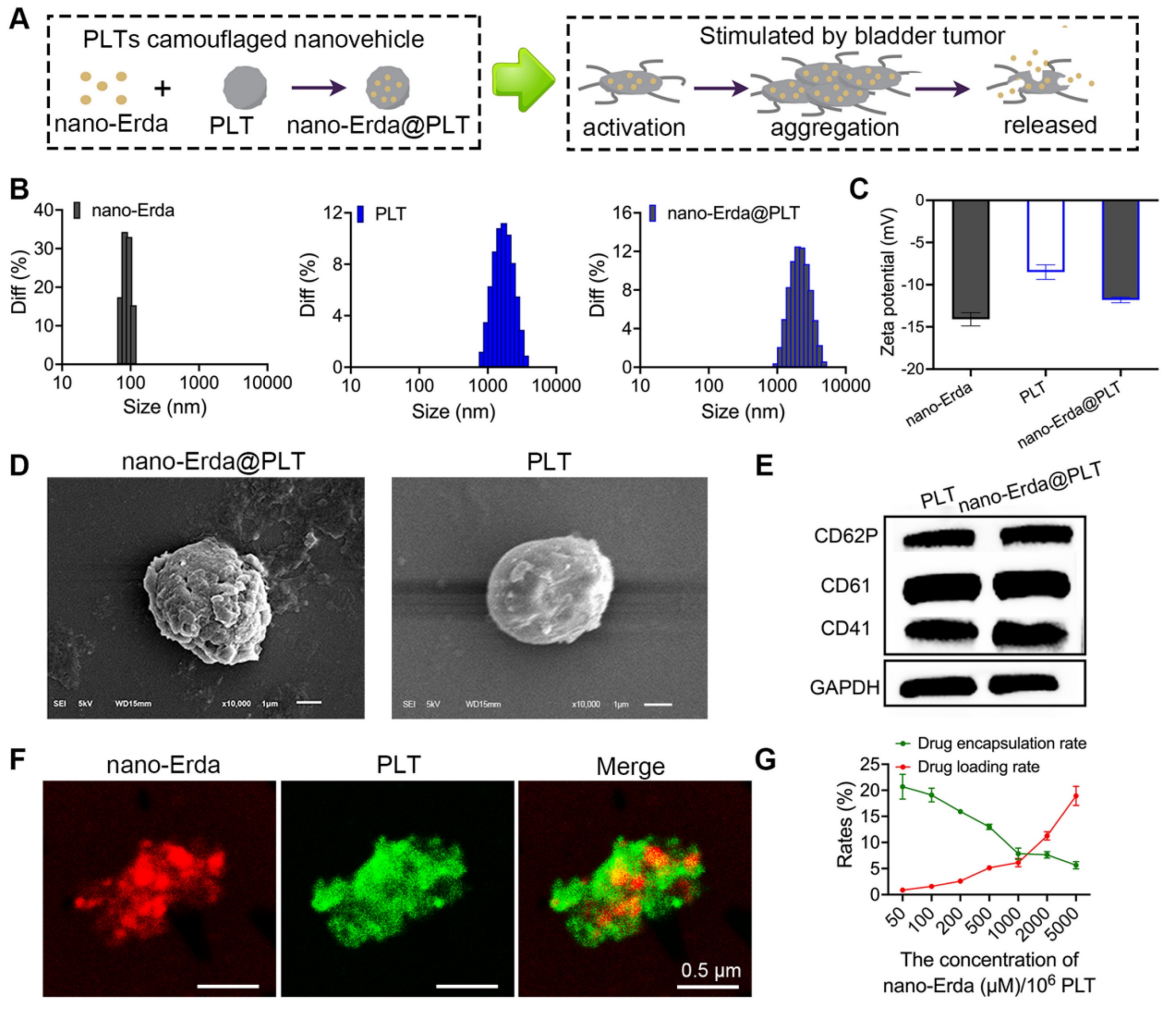
Construction and characterization of PLTs camouflaged nanovehicle for nano-Erda (nano-Erda@PLT). (A) Schematic diagram of nano-Erda@PLT and targeted delivery in bladder tumor. (B) Size distribution of nano-Erda, PLT, and nano-Erda@PLT. (C) The surface electrostatic potentials of nano-Erda, PLT, and nano-Erda@PLT. (D) The SEM images of nano-Erda@PLT and raw PLT. (E) WB results for raw PLT and nano-Erda@PLT. The key functional protein molecules CD41, CD61, and CD62p (p-selectin) of PLT were detected. (F) Photos of individual nano-Erda@PLT captured by confocal laser scanning microscopy. The nano-Erda was labeled with rhodamine fluorescence, while PLT was labeled with FITC fluorescence. (G) The drug loading rate and encapsulation rate of nano-Erda@PLT.

In this study, a nanovehicle camouflaged with PLTs was utilized as a carrier for nano-Erda to target drug delivery to the tumor site, with the goal of achieving drug accumulation in the tumor (Figure 2A). The initial step involved packaging nano-Erda into PLTs to construct nano-Erda@PLT. During this process, changes in nanoparticle size and surface potential were analyzed. The nano-Erda@PLT showed a slight increase in nanoparticle size compared to drug-free native PLTs, attributed to drug loading (Figures 2B and S6). As anticipated, the surface potential of nano-Erda@PLT closely resembled that of native PLTs, as illustrated in Figure 2C, indicating the presence of PLT membrane on the surface of nano-Erda. SEM was employed to assess the morphological alterations of nano-Erda@PLT (Figure 2D). The nano-Erda@PLT maintained a three-dimensional spherical shape with a rougher surface post drug encapsulation, without significant morphological changes, suggesting their inactivated state. PGE1 is a metabolite of arachidonic acid that inhibits PLT activation and aggregation 46. In this study, PGE1 was primarily utilized during the PLT extraction process to harness its inhibitory effects on PLT activation and aggregation. The PGE1 treatment would also enhance the stability of drug-loaded PLTs subsequently. As shown in Figure S7, nano-Erda@PLT exhibited good stability *in vitro*, maintaining its stable particle size for up to 6 days. To confirm the loading of PLTs on the surface of nano-Erda, the key functional proteins of PLTs, such as CD41, CD61, and CD62p (p-selectin) 47-49, were examined. As depicted in Figure 2E, the protein expression in nano-Erda@PLT mirrored that of raw PLTs, validating the presence of PLTs in the nano-Erda@PLT.

Furthermore, confocal laser scanning microscopy was utilized to characterize individual nano-Erda@PLT, as shown in Figure 2F. Each nano-Erda@PLT exhibited red fluorescence (rhodamine labeled nano-Erda) and green fluorescence (FITC labeled PLT). The merge image revealed that most of the red fluorescence was covered with green fluorescence, combining the results in Figure 2E, suggesting successful encapsulation of nano-Erda in PLTs and formation of nano-Erda@PLT.

Previous research has demonstrated that PLTs can effectively encapsulate various drugs, providing a versatile platform for drug delivery with high loading capacities 36,37. Ultrasound-mediated cavitation of microbubbles induces reversible poration of PLT membranes, a phenomenon known as sonoporation 50. This process transiently increases membrane permeability, enabling large molecular drugs that are normally excluded by the PLT membrane to enter the PLT interior through sonopores. Notably, PLTs can self-repair the sonopores within a short period, restoring normal membrane permeability and encapsulating the drug within the PLTs 51. Based on sonoporation, we hypothesized that nano-Erda particles could be loaded into PLTs. To investigate this, the drug loading rate of nano-Erda@PLT was investigated (Figure 2G), showing a linear relationship between drug loading and nanoparticle concentration. This indicates that increasing nanoparticle concentration results in a higher drug loading rate, while increasing the drug loading rate decreases the encapsulation efficiency of the samples. Considering cost and design requirements, a concentration of 1000 μM of nano-Erda in 10^6^ PLTs was selected for subsequent studies.

### Activation, targeting and accumulation of nano-Erda@PLT *in vitro* and *in vivo*

**Figure 3:**
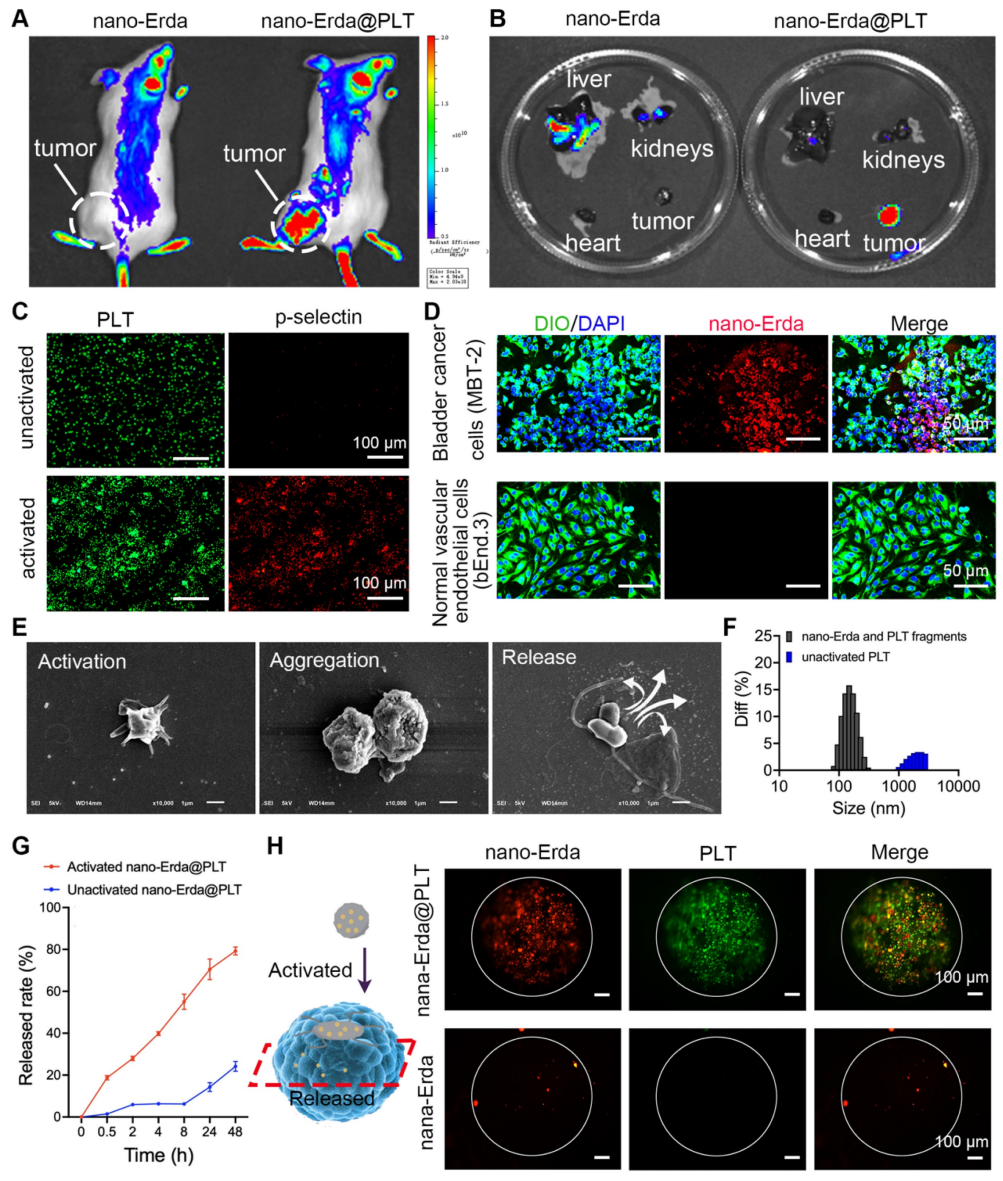
Targeting and accumulation of nano-Erda@PLT. (A) Live *in vivo* imaging results of bladder tumor bearing mice 0.5 h post-treatment. (B) *Ex vivo* fluorescence images 0.5 h post-treatment. (C) Immunofluorescence detection results of p-selectin expression before and after activation of nano-Erda@PLT. (D) Evaluation of tumor targeting and intravascular penetration of nano-Erda@PLT in the Transwell system. In the nano-Erda@PLT complex, the nano-Erda was labeled with rhodamine (red), the cell nuclei were stained with DAPI (blue), and the cell membranes were stained with DIO (green). (E) SEM images of the nano-Erda@PLT activation process, including activation, aggregation, and drug release. (F) Size distribution of the particles released from the activated nano-Erda@PLT. (G) The drug-releasing curve of nano-Erda@PLT with or without activators (ADP). (H) Schematic diagram of 3D tumor spheroids and photos captured by confocal laser scanning microscopy. Red fluorescence, rhodamine-labeled nano-Erda. Green fluorescence, FITC-labeled PLT.

In this study, PLTs were utilized as carriers for nano-Erda in order to facilitate targeted delivery of the nano-Erda to bladder cancer cells. Tumor-bearing ICR mice were randomly assigned to groups that either received nano-Erda@PLT administration or free nano-Erda administration. Through live *in vivo* imaging, it was observed that a substantial amount of fluorescent drugs accumulated in subcutaneous tumors just 0.5 hours after treatment with nano-Erda@PLT. In contrast, minimal fluorescence accumulation was detected in tumors following the injection of free nano-Erda (Figure 3A and S8). Furthermore, the total fluorescence values of Cy5.5 in the nano-Erda@PLT group were higher compared to those in the free nano-Erda group. It was postulated that PLTs extend the residence time of nano-Erda *in vivo* within the nano-Erda@PLT system by reducing drug clearance, possibly through a decrease in serum protein binding. In addition, the *ex vivo* fluorescence images of isolated organs were collected at 0.5 hours post-injection. Figure 3B and S9 depict efficient tumor accumulation in mice following the administration of nano-Erda@PLT. In contrast, nano-Erda distribution was primarily found in the liver and kidneys immediately after administration, attributed to capture and elimination by the reticuloendothelial system. *In vivo* fluorescence imaging illustrated that nano-Erda@PLT showed prolonged circulation time and targeted delivery to the bladder tumor.

ADP is known to be highly expressed in bladder cancer and has been reported to play a crucial role in activating platelet carriers 52,53. PLTs are recruited to the tumor microenvironment by various substances released by tumors, including ADP. Once recruited, PLTs become activated and exhibit the ability to internalize and secrete molecules, making them suitable for drug delivery applications 54,55. In this study, we utilized ADP to activate nano-Erda@PLT *in vitro*, mimicking the activation process of nano-Erda@PLT in bladder cancer. The activated nano-Erda@PLT exhibited increased levels of CD62p (p-selectin) (*P* < 0.0001), indicating enhanced tumor targeting capabilities post PLT activation, as demonstrated in the immunofluorescence images in Figure 3C and S10. These findings were consistent with the results from the Western blot experiment shown in Figure S11. Consequently, the nano-Erda@PLT remained inactive until reaching the bladder cancer site, allowing for prolonged circulation *in vivo*.

To further confirm the activation of nano-Erda@PLT in bladder tumors, a Transwell system was employed to evaluate its *in vitro* tumor-targeting ability (Figure S12). MBT-2 (mouse bladder cancer cells) or bEnd.3 (normal vascular endothelial cells) were placed in the lower chamber, while nano-Erda@PLT was added to the upper chamber. Following a 24-hour incubation period, the nano-Erda@PLT and migrated through the pores (0.4 μm) of the Transwell chamber into the lower chamber, selectively binding to the bladder cancer cells, with no drug fluorescence observed around the normal cells (Figure 3D). Fluorescence semi-quantitative analysis is depicted in Figure S13. To further demonstrate that the nano-Erda@PLT does not prematurely release the drug prior to targeting the tumor. After nano-Erda@PLT was administered to normal mice, there was no organ accumulation (Figure S14). This was likely due to the relative stability of nano-Erda@PLT (Figure S7) and the fact that normal cells do not activate it (Figure 3D). These results support the notion that nano-Erda@PLT remains inactive and circulates in the bloodstream until activated by bladder cancer cells.

Therefore, how does the activated nano-Erda@PLT pass through the blood vessels to target the tumor? The particle size of nano-Erda@PLT exceeding 1 μm, as depicted in Figure 1C, direct passage through the 0.4 μm gap in the Transwell system was initially deemed impossible. This limitation prompted a study on the activated release of nano-Erda@PLT. To elucidate the deformation behavior of nano-Erda@PLT, we investigated the process of nano-Erda@PLT activation and drug release in bladder cancer vasculature and tumor cells* in vitro*. Upon the addition of ADP into nano-Erda@PLT, rapid activation and release of internal drugs were observed, as shown in Figure 3E. The activated nano-Erda@PLT changed from a spherical shape to a spindle shape and extended multiple pseudopods. Immediately after, the activated nano-Erda@PLT particles further aggregated with other nano-Erda@PLT and eventually released the nano internal drugs. Subsequently, the particle size of activated nano-Erda@PLT and their released drugs was measured, as depicted in Figure 3F. Analysis revealed that the size of released particles from nano-Erda@PLT was approximately 100 nm, facilitating their passage through blood vessels to reach the tumor.

During the activation of nano-Erda@PLT, the drug release rate was monitored. Figure 3G illustrates that the activated nano-Erda@PLT rapidly released a substantial amount of drugs, with the majority of the encapsulated drugs being released within the observation period. In contrast, the inactivated nano-Erda@PLT maintained a stable state over an extended period and only released a small amount of drugs within the 48-hour observation window.

Traditional monolayer cell culture lacks the ability to simulate the tumor microenvironment* in vivo*. However, 3D tumor spheroids can accurately mimic specific characteristics of solid tumors, such as tumor tissue permeability. In this study, 3D tumor spheroids were utilized to further showcase the tumor targeting and penetration of nano-Erda@PLT (Figure 3H and S15). Fluorescently labeled nano-Erda@PLT (containing rhodamine-labeled nano-Erda and FITC-labeled PLT) or free rhodamine-labeled nano-Erda were introduced to the 3D tumor spheroid cell culture dish. After 12 hours, laser confocal microscopy was employed to observe the penetration of nano-Erda@PLT within the tumor tissues. The fluorescence of PLT and nano-Erda was detected at the center of the spheroid in the nano-Erda@PLT group, while only a small amount of nano-Erda was visible inside the spheroid in the free nano-Erda treated group. This phenomenon may be attributed to the fact that nano-Erda@PLT specifically targets tumors with the assistance of PLTs, leading to an increased concentration on the tumor surface. Additionally, the nano-Erda surface released by activated nano-Erda@PLT is coated with a PLT membrane, enhancing the tumor's recognition and internalization of nano-Erda, consequently achieving a high concentration and deep penetration within the tumor.

Based on the above results, we surmise that nano-Erda@PLT could circulate in the bloodstream for an extended duration before ultimately localizing to tumor tissues. Then activated by cytokines, such as ADP released from tumor tissues, nano-Erda@PLT undergoes an aggregation reaction, subsequently releasing nano-Erda particles into the tumor tissues through the imperfect tumor vascular wall. This process is feasible because the vascular wall in tumors is imperfect, allowing substances smaller than 1 μm to leak.

### *In vitro* results of nano-Erda@PLT induced pyroptosis

**Figure 4:**
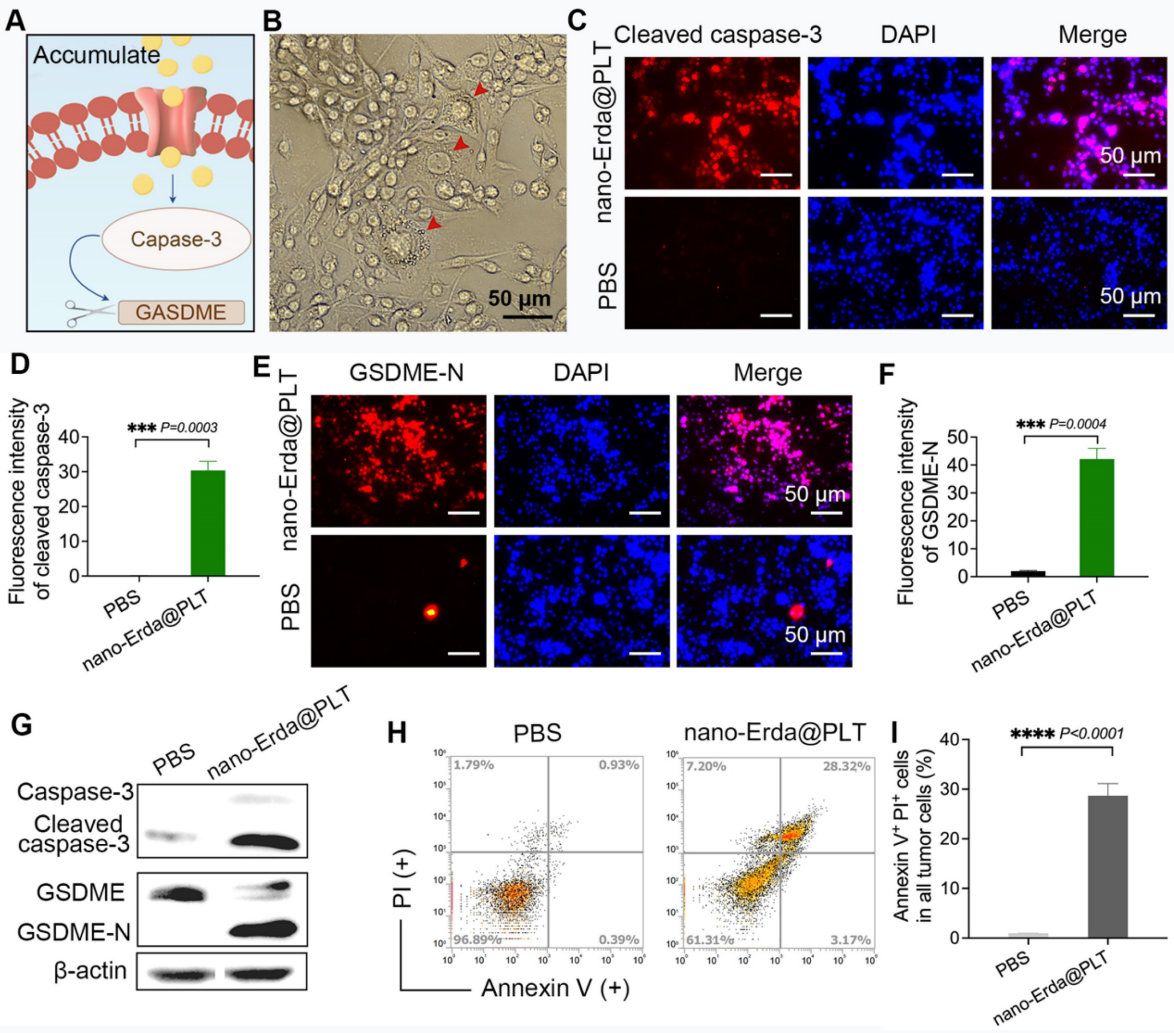
The pyroptotic effect of nano-Erda@PLT on MBT-2 cells *in vitro*. (A) A schematic representation of the pyroptosis (capase-3/GSDME pathway) after nano-Erda@PLT treatment. (B) Representative pictures of pyroptosis after nano-Erda@PLT treated. Red arrows indicated cell swelling, rupture, content release, vacuole formation, etc. (C) Immunofluorescence results of cleaved caspase-3 in nano-Erda@PLT (with an effective concentration of 20 μM of Erda) treated group and PBS. (D) Semi-quantitative analysis of cleaved caspase-3. (E) Immunofluorescence detection results of GSDME-N. (F) Semi-quantitative analysis result of GSDME-N. (G) WB results of key pyroptotic molecules, including caspase-3, cleaved caspase-3, GSDME and GSDME-N. (H) Representative flow cytometry results of nano-Erda@PLT treated MBT-2 cells. (I) Viability (% of Annexin V^+^ PI^+^ cells). ****Significantly different compared to control (*P* < 0.0001).

Previous results have demonstrated that inducing pyroptosis in bladder cancer cells requires the accumulation of nano-Erda within tumors, with the PLT carrier being capable of effectively delivering drugs to bladder cancer cells. Then, we further investigated the induction of pyroptosis in bladder cancer cells using nano-Erda@PLT, focusing on the caspase-3/GSDME pathway (Figure 4A). Following 24 hours of nano-Erda@PLT treatment, typical morphological features of pyroptosis were observed in bladder cancer cells, including cell swelling, rupture, content release, and vacuole formation (Figure 4B).

Immunofluorescence tests for cleaved capase-3 and GSDME-N confirmed the activation of the capase-3/GSDME pathway by nano-Erda@PLT (Figure 4C-F). The fluorescence intensity for cleaved caspase-3 was significantly higher in bladder cancer cells treated with nano-Erda@PLT* in vitro* compared to the PBS control group (Figure 4C), as supported by semi-quantitative analysis (*P* = 0.0003) (Figure 4D). Similar results were observed for GSDME-N, with significant differences confirmed through immunofluorescence and semi-quantitative analysis (*P* = 0.0004) (Figure 4E-F). Successively, it showed a significant increase in cleaved caspase-3 and GSDME-N levels from the Western blot analysis results (Figure 4G). Flow cytometry analysis revealed a notable increase in Annexin-V^+^ PI^+^ cells in bladder cancer cells treated with nano-Erda@PLT compared to PBS treatment (*P* < 0.0001) (Figure 4H-I).

Combined with the results in Figure 4, those enabled us to draw the conclusion that nano-Erda@PLT treatment of bladder cancer cells promoted cell pyroptosis. This was evidenced by the appearance of pyroptosis-specific swelling morphology, increased cleaved caspase-3, and GSDME-N. Flow cytometry results also further demonstrated that nano-Erda@PLT had the ability to promote pyroptosis in bladder cancer cells i*n vitro*.

### The immunotherapy efficacy of nano-Erda@PLT *in vivo*

**Figure 5:**
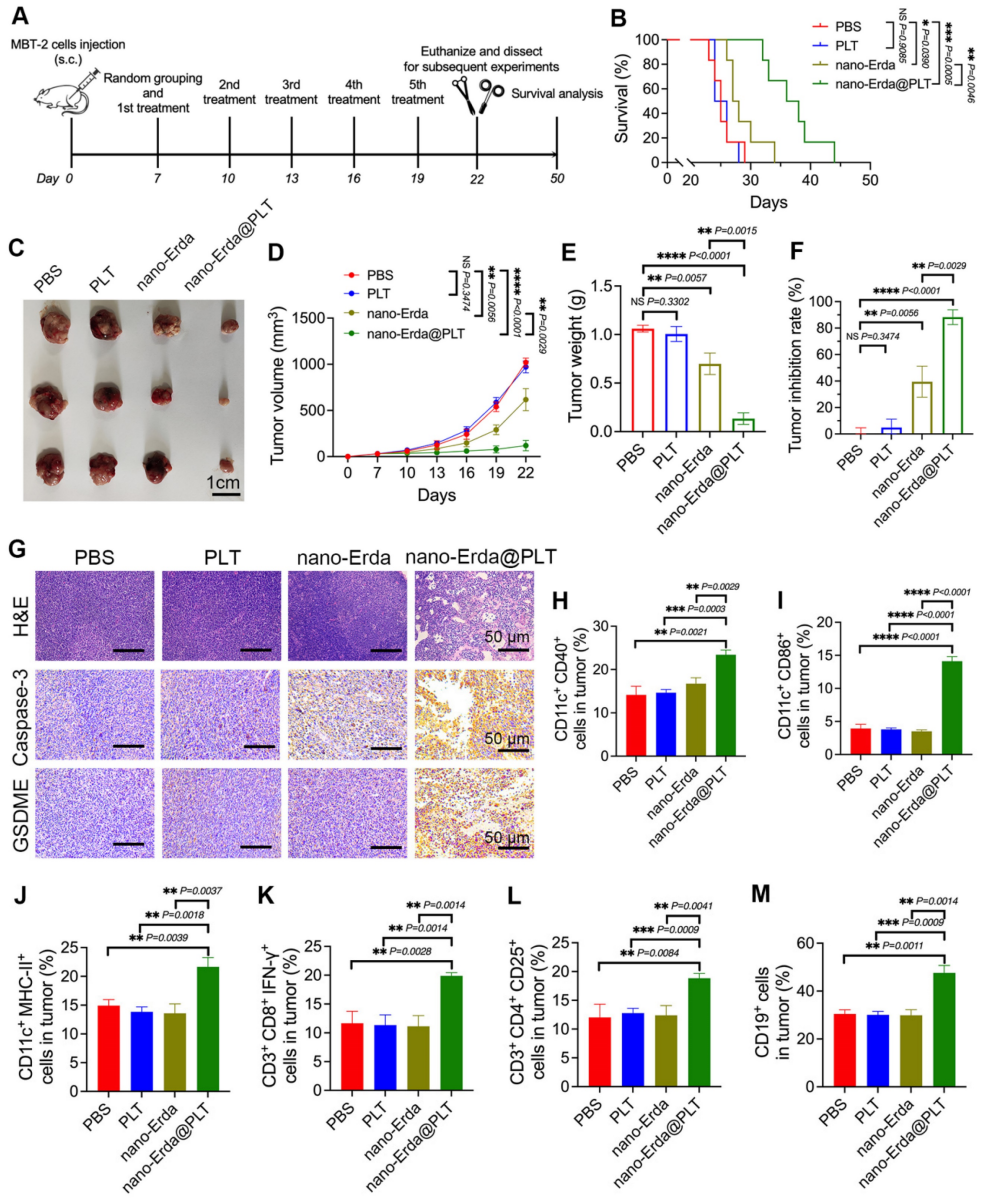
Evaluation of the antitumor effects and the immunotherapy efficacy of nano-Erda@PLT in a subcutaneous bladder cancer model. (A) Schema showing the timeline of model construction and intravenous injection. Following the subcutaneous injection of MBT-2 cells in C3H mice, the mice were randomly divided into four groups and received specific treatments (PBS, PLT, nano-Erda, or nano-Erda@PLT) on days 7, 10, 13, 16, and 19. (B) The survival curves of each group of mice. (C) Images of the MBT-2 tumors at day 22 post administration with different treatments. (D) Volume growth curves of subcutaneous tumors in each group of mice. (E) Comparison of tumor weights in each group of mice. (F) Comparison of tumor inhibition rates in each group of mice. (G) H&E staining of subcutaneous tumor tissue sections and immunohistochemical examination of caspase-3 and GSDME in each group of mice. (H-M) Flow cytometry analysis of immune cells in tumor tissues of each group of mice, including: CD11c^+^CD40^+^ cells (H), CD11c^+^CD86^+^ cells (I), CD11c^+^MHC-II^+^ cells (J), CD3^+^CD8^+^IFN-γ^+^ cells (K), CD3^+^CD4^+^CD25^+^ cells (L), and CD19^+^ cells (M). *Significantly different compared to control (*P* < 0.05). **P < 0.01, ***P < 0.001, ****P < 0.0001.

The nano-Erda@PLT was shown to effectively induce pyroptosis *in vitro* in bladder cancer cells, prompting our investigation into its potential to induce pyroptosis in bladder tumor-bearing model mice. The treatment process of the mice is illustrated in Figure 5A. After subcutaneous injection of MBT-2 cells in C3H mice, the mice were randomly divided into four groups and received specific treatments (PBS, PLT, nano-Erda, or nano-Erda@PLT) on days 7, 10, 13, 16, and 19. To respect ethical animal experimentation, the animals were euthanized on day 22 when the tumor burden of the control groups became excessive. At day 22, three mice per group were euthanized, and their subcutaneous tumors were dissected and weighed. The remaining six mice in each group were monitored until death or until their tumor volume reached the ethical standards for animal welfare. Euthanasia was performed when the tumor volume of any mouse met the ethical standards for animal welfare.

As we expected, the mice treated with nano-Erda@PLT showed a mean overall survival of 37.0 ± 4.38 days, while those receiving PBS, PLT, or nano-Erda had mean overall survival times of 25.3 ± 2.07, 25.3 ± 1.63, and 28.7 ± 2.94 days, respectively (Figure 5B). In Figure 5C-F, it is evident that the nano-Erda@PLT group displayed a more pronounced inhibition of tumor growth compared to the other groups. Tumor collection on day 22 (Figure 5C) revealed that the nano-Erda@PLT group had the lowest tumor volume and weight among all groups (Figure 5D-E). The tumor inhibition rate (Figure 5F), calculated based on the tumor volume at the endpoint, was significantly higher in the nano-Erda@PLT group (88.31 ± 4.216%), compared to the PBS group, than the nano-Erda group (39.49 ± 7.285%). This difference was statistically significant (*P* = 0.0029).

The tumor tissues of mice were analyzed using H&E staining and immunohistochemical examination to evaluate the therapeutic effects of nano-Erda@PLT. Results from Figure 5G showed a significant regression of tumor cells in response to treatment in the nano-Erda@PLT group, with tumor cells degenerating and being replaced by collagenized, fibrotic tissue, or tissue macrophages. Immunohistochemical analysis revealed strong positivity for caspase-3 and GSDME in tumor cells of the nano-Erda@PLT group, while cells in other groups showed negative or weak positivity for these markers (Figure 5G). These findings suggest that nano-Erda@PLT has the potential to induce pyroptosis in bladder cancer cells, showcasing promising anti-tumor effects in treating bladder cancer in mice.

Pyroptosis has been increasingly recognized for its role in regulating immune responses to cancer. Although nano-Erda@PLT can induce apoptosis in tumor cells, apoptosis is a non-inflammatory form of cell death that typically results in immune silence and subsequent inhibition of immune responses 56. In contrast, pyroptosis is a pro-inflammatory form of cell death characterized by the rapid rupture of the cell membrane and the release of numerous inflammatory molecules, leading to a robust immune response 57,58. As our understanding of the complex interplay between pyroptosis pathways and the immune system grows, it is becoming evident that pyroptotic cell death can actively engage and modulate antitumor immune responses. Then, we investigated the efficacy of immunotherapy of nano-Erda@PLT.

Dendritic cells (DCs) play a crucial role in antigen presentation, particularly in activating cytotoxic T cells (CTLs) in immune responses. CD40 and CD86 are considered co-stimulatory markers of DCs, while MHC-II is a surface activation marker of DCs. In the nano-Erda@PLT group, CD11c^+^CD40^+^ cells, CD11c^+^CD86^+^ cells, and CD11c^+^MHC-II^+^ cells were significantly increased, indicating that the treatment with nano-Erda@PLT promoted the activation of DCs within the tumor microenvironment and facilitated the presentation of tumor antigens to CTLs (Figure 5H-J). CD3^+^CD8^+^IFN-γ^+^ T cells are recognized as CTLs capable of killing tumor cells and other antigenic substances. The nano-Erda@PLT group exhibited a significant increase in CD3^+^CD8^+^IFN-γ^+^ cells compared to the other groups, suggesting the significant activation of CTLs and their anti-tumor effect (Figure 5K). CD3^+^CD4^+^CD25^+^ T cells are regulatory T cells (Tregs) that play an immunoregulatory role in immune responses, and CD19 is a surface marker of B cells (Figure 5L-M). The nano-Erda@PLT group also showed a significant increase in CD3^+^CD4^+^CD25^+^ cells and CD19^+^ cells, indicating that the constructed nano-Erda@PLT stimulated both cellular and humoral immunity.

### The biosafety of nano-Erda@PLT

Ensuring the biocompatibility and biosafety of medical materials is crucial to protect patient health. Biocompatibility refers to a material's ability to interact with the biological system without causing harm, while biosafety involves assessing and managing potential risks. In this study, the biosafety of nano-Erda@PLT was evaluated. The *in vitro* biosafety results (Figure S16) demonstrated that upon encapsulation by PLT, nano-Erda@PLT exhibited significantly enhanced biocompatibility and reduced cytotoxicity towards normal cells. This improved biocompatibility is attributed to the reduced direct interaction between nano-Erda and vascular cells due to the protective barrier provided by the PLT coating.

**Figure 6:**
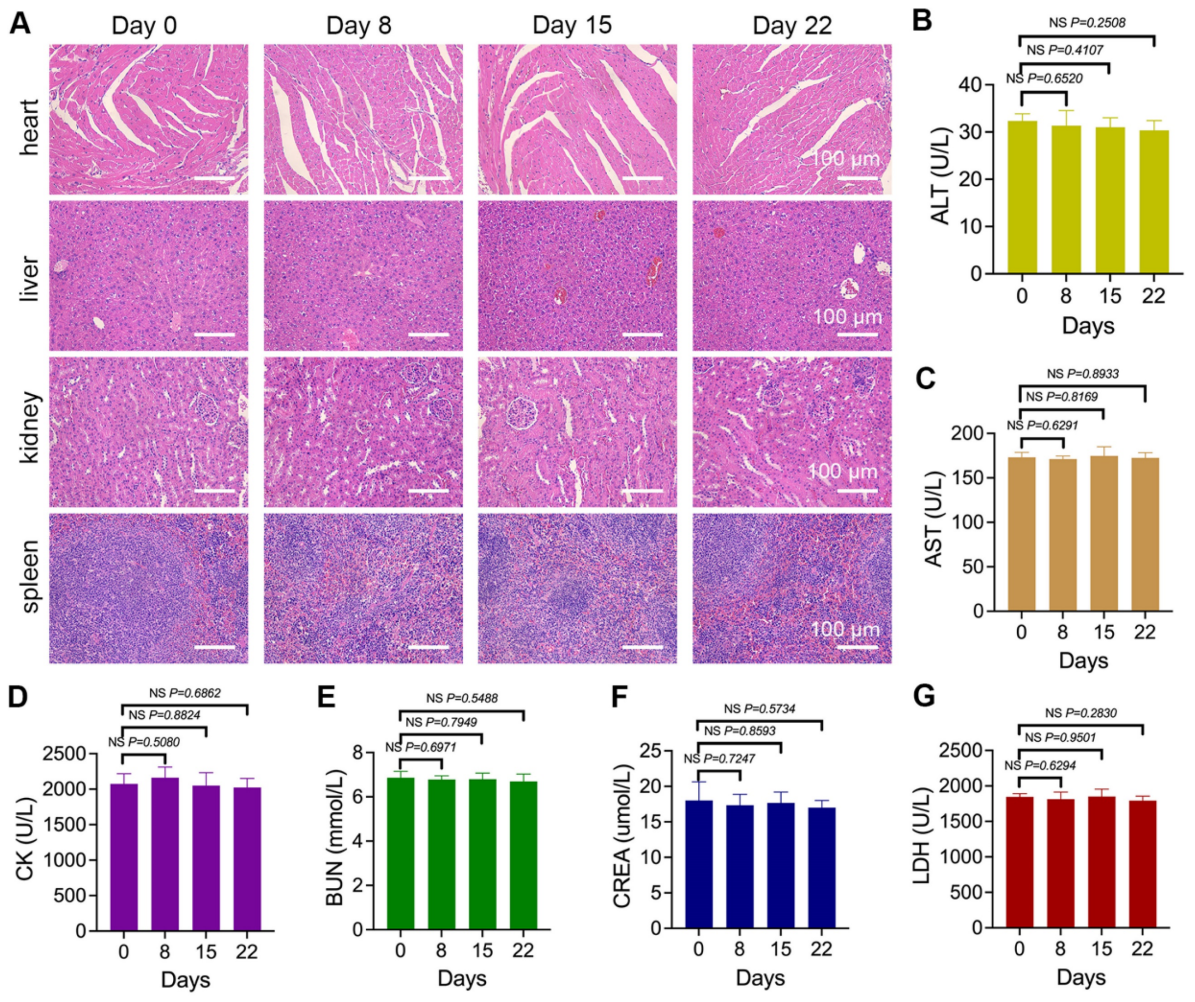
The biosafety results of nano-Erda@PLT. (A) Microscopic images of H&E-stained sections of vital organs including the hearts, livers, kidneys, and spleens of tumor-bearing mice treated with nano-Erda@PLT for 0, 8, 15 and 22 days. (B-G) Biochemical analysis of serum biomarkers in the peripheral blood of the mice, including ALT (B) reflecting liver function, AST (C) and CK (D) reflecting myocardial injury, BUN (E) and CREA (F) reflecting kidney function, and LDH (G) reflecting systemic organ tissue damage. NS = not significant.

Mice were treated with nano-Erda@PLT for 8, 15, and 22 days, followed by organ dissection. Histological analysis of the heart, liver, kidneys, and spleen showed normal microscopic morphology with no significant toxic reactions compared to pre-treatment (Figure 6A). Throughout the treatment, peripheral blood was periodically collected for serum biomarker analysis, including ALT reflecting liver function, AST and CK reflecting myocardial injury, BUN and CREA reflecting kidney function, and LDH reflecting systemic organ tissue damage. As shown in Figure 6B-G, no statistically significant differences were observed in biomarkers during the entire treatment period.

Overall, it is preliminarily considered that the constructed nano-Erda@PLT demonstrated good biosafety within the experimental dosage range, showing no significant damage to vital organs in mice. These findings suggest that the nano-Erda@PLT is well tolerated and non-toxic.

## Conclusions

In this study, a PLTs-camouflaged nanovehicle was developed successfully for delivering nano-Erda to induce tumor pyroptosis for immunotherapy. The biotropism of PLTs towards the post-operative inflammatory microenvironment facilitated efficient tumor delivery through PLT hitchhiking. The nano-Erda@PLT nanovehicle exhibited strong tumor targeting, transvascular effect, and tumor permeability, while maintaining stability and rapid activation. Through the caspase-3/GSDME pathway, nano-Erda@PLT accumulation induced pyroptosis in bladder cancer cells, leading to the release of tumor antigens and inflammatory factors. This activation of the immune system, particularly DCs and T cells, resulted in the attack on bladder cancer tissues and eventual tumor clearance. Furthermore, nano-Erda@PLT demonstrated good biosafety. This research offers a promising and efficient approach to tumor immunotherapy and provides foundational data for the potential clinical application of PLTs camouflaged nanovehicles.

## Supplementary Material

Supplementary figures and tables.
