# Supplementary material for: Platelets camouflaged nanovehicle improved bladder cancer immunotherapy by triggering pyroptosis
Source: Theranostics. 2024 Oct 14;14(17):6692–707. doi: 10.7150/thno.99040 (PMC11519802; doi:10.7150/thno.99040)
Supplement: Supplementary file 1 — Supplementary figures and tables. [file thnov14p6692s1.pdf]

Supporting Information

Platelets camouflaged nanovehicle improved bladder cancer immunotherapy by triggering pyroptosis

Jiale Tian [1*], Mingde Gao [1*], Jinfeng Zhu [1], Haifei Xu [1], Hao Ji [1], Donglin Xia [1#], Xiaolin Wang [1#]

1. Nantong Tumor Hospital, Affiliated Tumor Hospital of Nantong University & School of Public Health of Nantong University, Nantong, 226000, P. R. China

*These authors contributed equally.

[#] Corresponding authors at:

E-mail addresses: xiadonglin@ntu.edu.cn (D.L Xia), cxhwyc2010@163.com (X.L Wang)

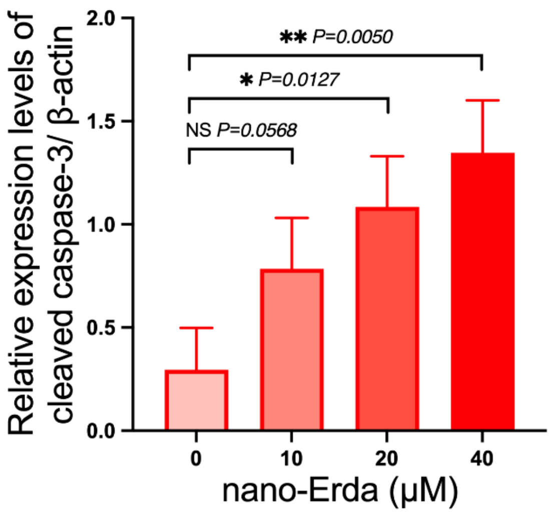

Fig. S1. Quantitative analysis of Western blot bands for cleaved caspase-3 protein in the pyroptosis pathway (at nano-Erda drug concentrations of 0, 10, 20, and 40 μM). Significant statistical differences in the expression of cleaved caspase-3 protein were observed between the control group and the groups treated with nano-Erda concentrations of 20 and 40 μM.

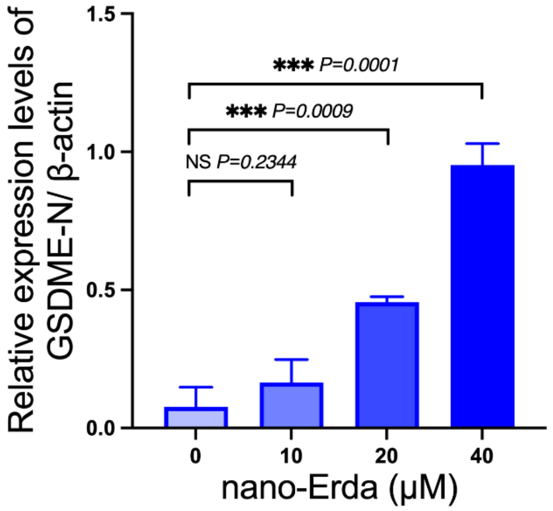

Fig. S2. Quantitative analysis of Western blot bands for GSDME-N protein in the pyroptosis pathway (at nano-Erda drug concentrations of 0, 10, 20, and 40μM). Significant statistical differences in the expression of GSDME-N protein were observed between the control group and the groups treated with nano-Erda concentrations of 20 and 40 μM.

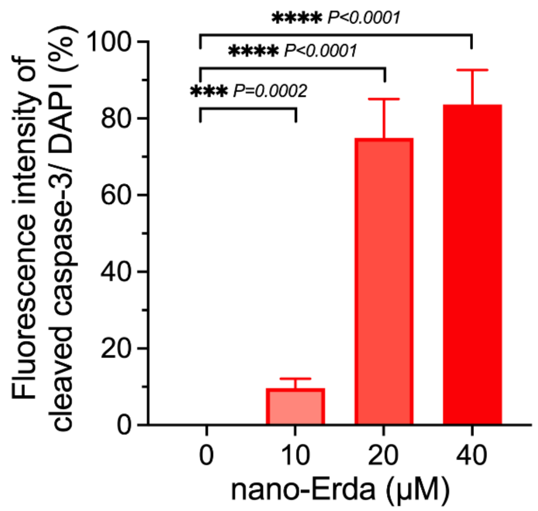

Fig. S3. Immunofluorescence semi-quantitative analysis of cleaved caspase-3 in the pyroptosis pathway. Significant statistical differences in the fluorescence expression of caspase-3 molecule were observed between the control group and the groups treated with nano-Erda concentrations of 10, 20, and 40 μM.

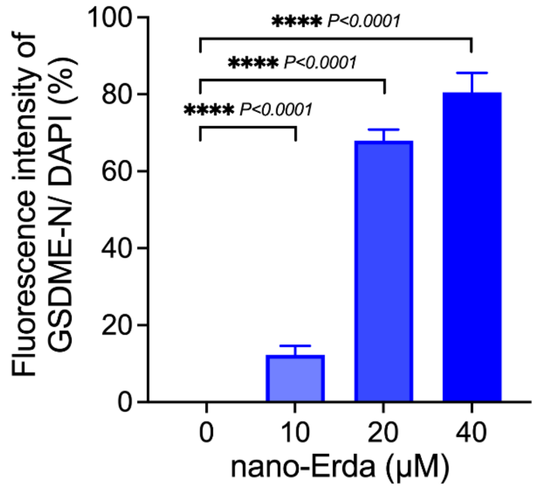

Fig. S4. Immunofluorescence semi-quantitative analysis of GSDME-N in the pyroptosis pathway. Significant statistical differences in the fluorescence expression of GSDME molecule were observed between the control group and the groups treated with nano-Erda concentrations of 10, 20, and 40 μM.

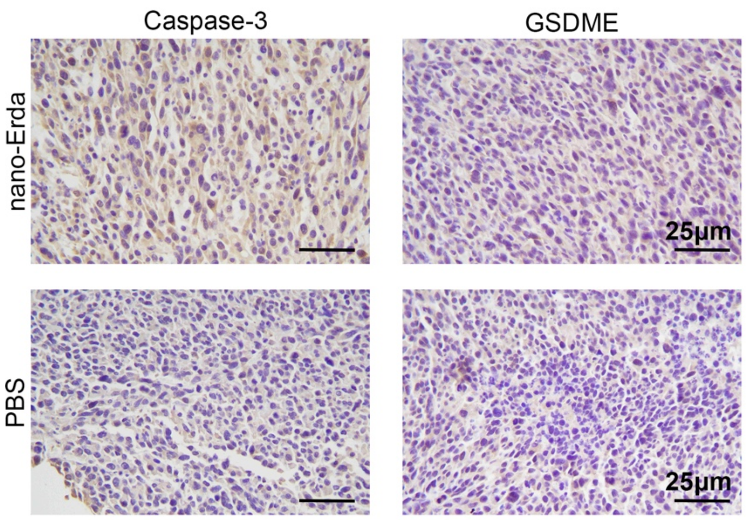

Fig. S5. Immunohistochemical results of caspase-3 and GSDME in tumors of mice treated with nano-Erda and PBS for 5 times (with 300 nmol of nano-Erda per injection).

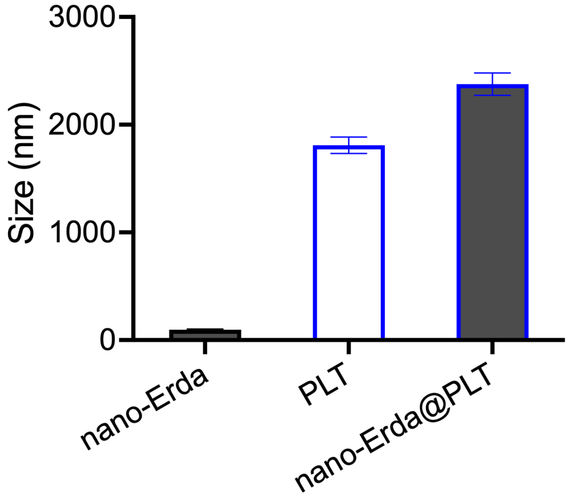

Fig. S6. The average nanoparticle sizes of nano-Erda, PLT and nano-Erda@PLT, which were 96.4±7.10 nm, 1808.5±75.95 nm, and 2376.6±104.37 nm, respectively.

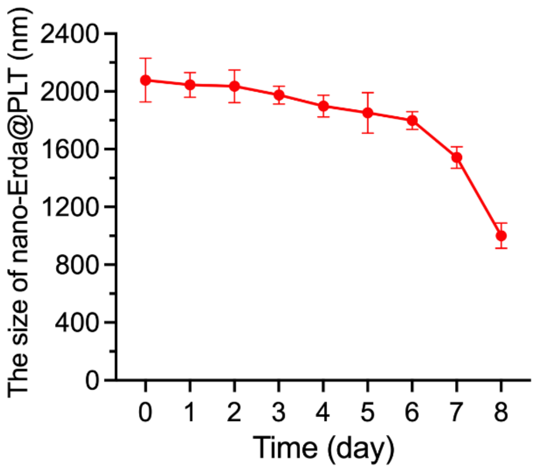

Fig. S7. The particle size of nano-Erda@PLT was measured over multiple days. The results indicated the stable particle size throughout the observation period, especially in the first 6 days, suggesting that the system did not undergo activation.

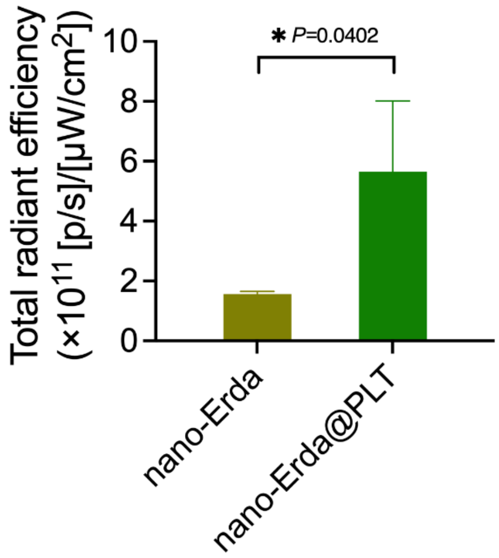

Fig. S8. Quantitative statistics of fluorescence in subcutaneous tumors in mice's live imaging experiments. The drug fluorescence levels of nano-Erda@PLT were significantly higher than those of free nano-Erda in subcutaneous bladder cancer in mice, providing quantitative evidence.

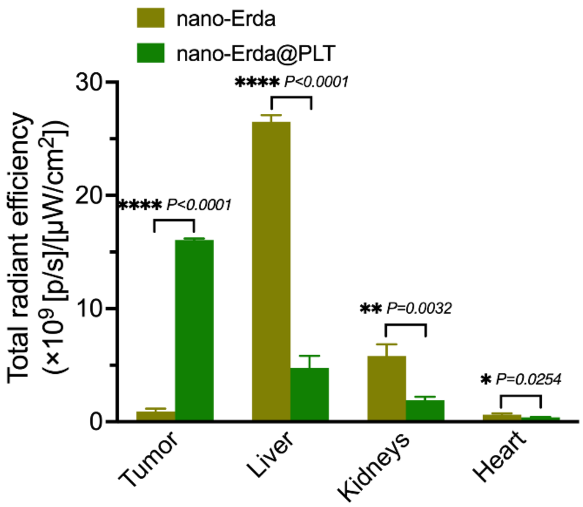

Fig. S9. Quantitative statistics of fluorescence in vital organs and tumors was conducted after dissecting the mice used in the in vivo imaging experiment. Compared to the mice in the free nano-Erda group, the mice in the nano-Erda@PLT group exhibited a significant increase in drug fluorescence content in subcutaneous tumors, a significant decrease in drug fluorescence content in the liver and kidneys. This suggests that nano-Erda@PLT effectively targets and delivers more drugs to the interior of the tumor, while reducing metabolism in the liver and kidneys. * $P < 0.05$, ** $P < 0.01$, ****$P < 0.0001$.

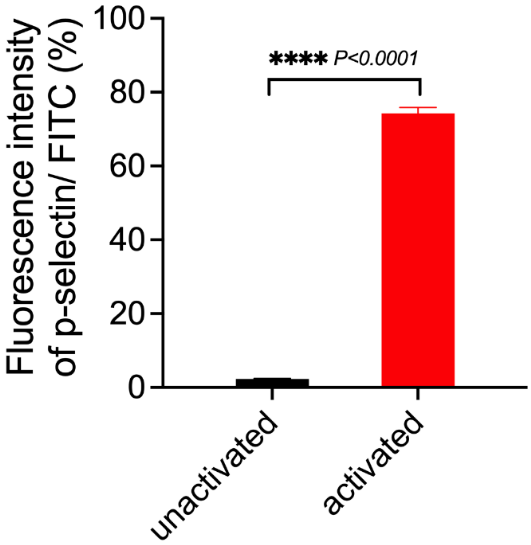

Fig. S10. Semi-quantitative analysis of P-selectin fluorescence was performed before and after activation of nano-Erda@PLT. The activated nano-Erda@PLT exhibited significantly higher expression of P-selectin, indicating strong tumor-targeting capabilities.

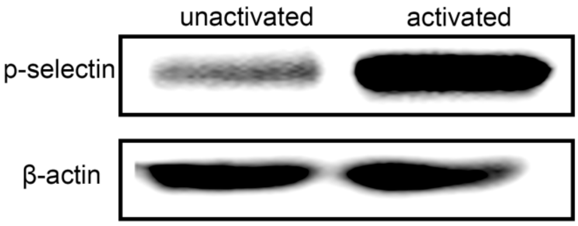

Fig. S11. The Western blot results of P-selectin protein before and after activation of nano-Erda@PLT confirmed that the activated nano-Erda@PLT had significantly higher levels of P-selectin molecules protein.

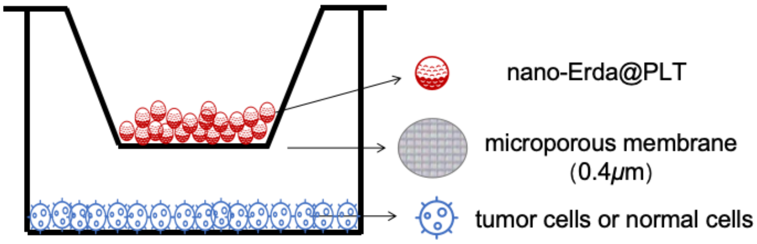

Fig. S12. A schematic diagram of the tumor-targeting in vitro cell experiment using the Transwell (cell migration and invasion assay) technique. In the diagram, nano-Erda in nano-Erda@PLT was labeled with rhodamine (red) fluorescence. The MBT-2 mouse bladder cancer cells and bEnd.3 mouse normal endothelial cells were cultured in the lower chamber, which was separated from the upper chamber by a Transwell insert with a 0.4 µm microporous membrane. Nano-Erda@PLT were added to the upper chamber. After 24 hours of incubation, the Transwell inserts were removed, and the nuclei of the MBT-2 cells and bEnd.3 cells were stained with Hoechst33342 (blue) fluorescence. The fluorescent distribution of nano-Erda in the surrounding area of the lower layer cells was observed.

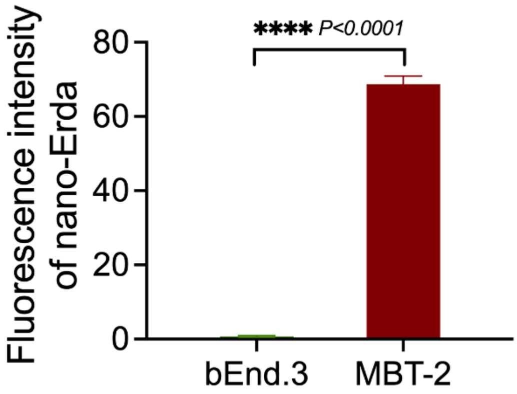

Fig. S13. Semi-quantitative analysis of nano-Erda drug fluorescence was conducted in the in vitro tumor-targeting cell experiment using the Transwell mechanism. The nano-Erda fluorescence observed around MBT-2 mouse tumor cells was significantly higher than that around bEnd.3 normal cells, and this difference was statistically significant.

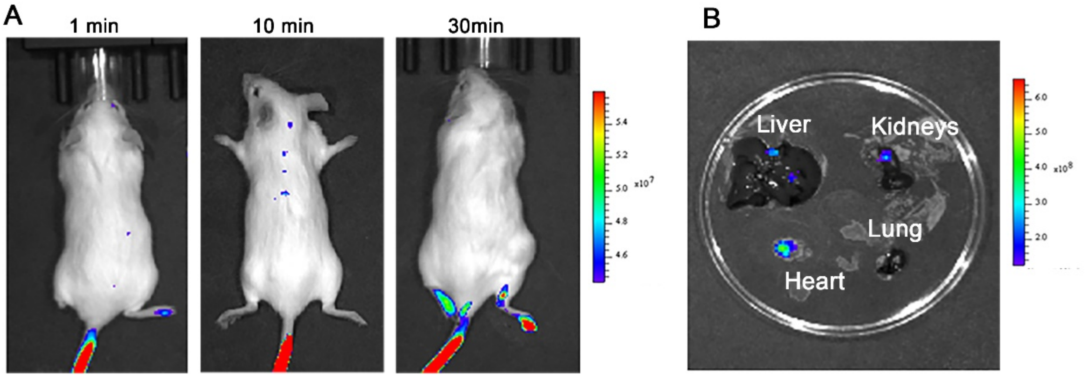

Fig. S14. The stability of nano-Erda@PLT *in vivo*. (A) Live *in vivo* imaging results of normal mice 0.5 h post-treatment. (B) Ex vivo fluorescence images 0.5 h post-treatment. After nano-Erda@PLT was administered to normal mice, there was no organ accumulation. It seems reasonable to speculate that nano-Erda@PLT does not prematurely release the drug prior to targeting the tumor.

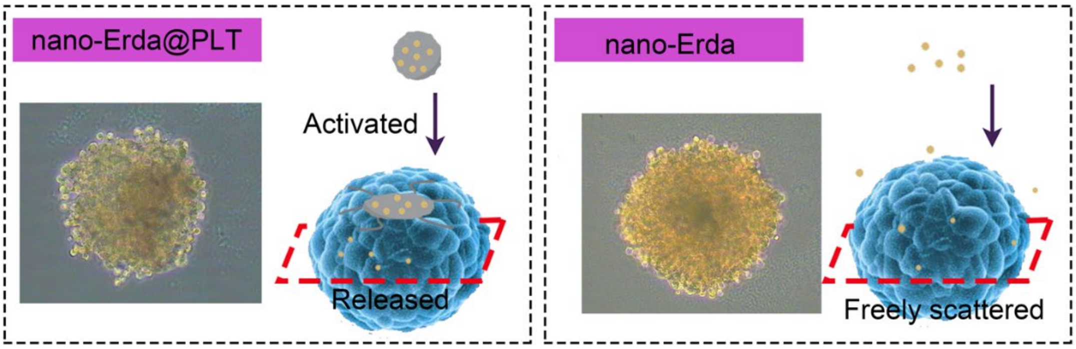

Figure S15. 3D tumor spheroid images and schematic diagram of nano-Erda@PLT or nano-Erda in 3D tumor spheroids. Nano-Erda@PLT could specifically target to tumors with the assistance of PLTs, leading to an increased concentration on the tumor surface. The nano-Erda is freely dispersed in solution and cannot form a high concentration on the tumor surface.

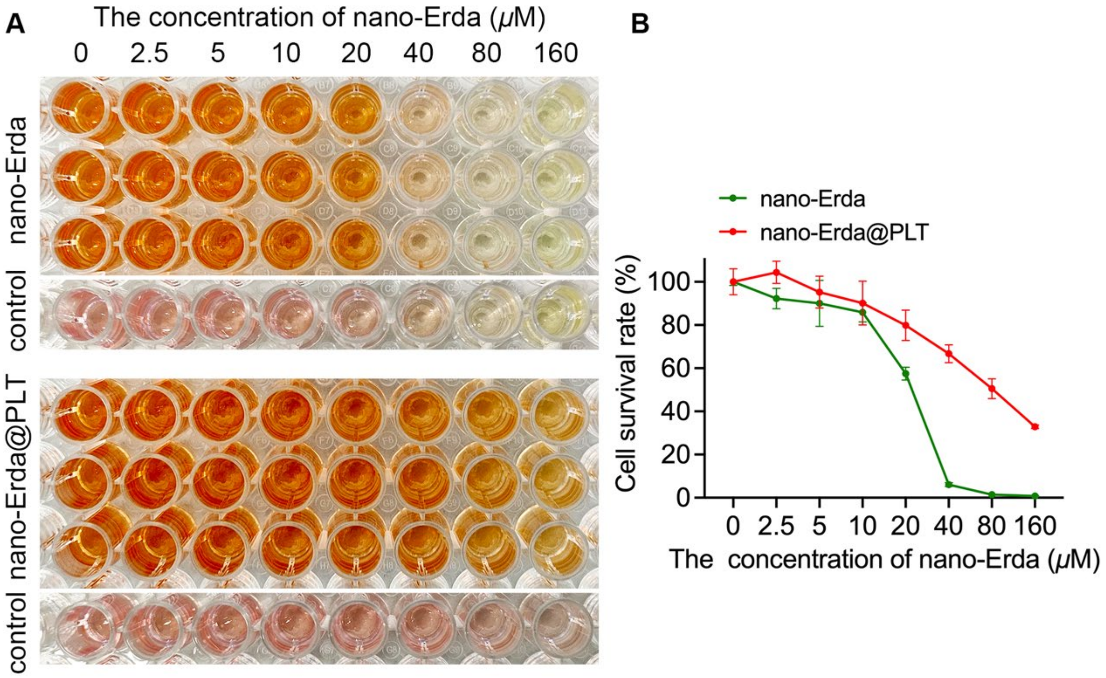

Fig. S16. The result of the cytotoxicity assay. The effect of nano-Erda@PLT on bEnd.3 cells was evaluated using a CCK-8 assay. The results demonstrated that nano-Erda@PLT exhibited significantly reduced toxicity towards bEnd.3 cells, compared to nano-Erda, at the same concentration.
